# Gynecologists are afraid of prescribing hormone replacement to endometrial/ovarian cancer survivors despite national guidelines—a survey in Sweden

**DOI:** 10.1080/03009734.2018.1544597

**Published:** 2018-12-10

**Authors:** Sandra Halldorsdottir, Hanna Dahlstrand, Karin Stålberg

**Affiliations:** a Department of Women’s and Children’s Health, Uppsala University, Uppsala, Sweden;; b Department of Immunology, Genetics and Pathology, Uppsala University, Uppsala, Sweden

**Keywords:** Endometrial cancer, hormone replacement therapy, ovarian cancer, survey

## Abstract

**Background:** Prolonged survival in ovarian and endometrial cancer patients increases the importance of paying attention to quality of life. Hormone replacement therapy (HRT) after gynecologic cancer has been controversial. With this survey, we sought to describe Swedish gynecologists’ and gynecologic oncologists’ attitudes towards prescribing HRT to these cancer survivors and see if prescribing practice is consistent with the available evidence and national guidelines.

**Material and methods:** A web-based survey containing three hypothetical cases with a total of 15 questions was distributed to gynecologists and gynecologic oncologists in Sweden. Respondents were asked about their HRT prescription practices in endometrial/ovarian cancer patients with moderate to severe menopausal symptoms.

**Results:** In total 262 gynecologists and 24 gynecologic oncologists answered the survey. In the low-risk endometrial cancer case a majority of the gynecologists (55%) and gynecologic oncologists (66.7%) would prescribe local estrogen. A total of 30% of the gynecologists would prescribe estrogen replacement therapy (ERT) in the high-risk endometrial cancer case compared to 58.3% of the gynecologic oncologists. The gynecologic oncologists felt more comfortable treating patients with endometrial cancer than did gynecologists, and the gynecologists were more likely to read the national guidelines. In the ovarian cancer case, 63.7% of the gynecologists would prescribe HRT compared to 92% of the gynecologic oncologists.

**Conclusion:** Swedish gynecologic oncologists have a more favorable attitude towards HRT for endometrial/ovarian cancer patients and feel more comfortable treating their patients than do gynecologists. This study illustrates a need for education in these matters in order not to withhold HRT from women due to doctors’ sometimes unjustified anxiety.

## Introduction

Symptoms of iatrogenic menopause are usually considerably more severe in comparison to those following a naturally occurring menopause and might adversely affect the quality of life in young female cancer survivors (1). Progress in the treatment of gynecological cancer has given many women prolonged survival, and therefore it is increasingly important to pay attention to long-term consequences of estrogen deficiency. The most effective treatment for menopausal symptoms is hormone replacement therapy (HRT). HRT is effective for the treatment of vasomotor symptoms, preventing osteoporosis, reducing the risk of cardiovascular disease (in women <60 years old), perimenopausal low mood, sexual dysfunction, and urogenital symptoms (2–4).

Endometrial cancer is the most common gynecological malignancy in Sweden, and its incidence has increased over the last decades from 18.5/100,000 persons/year in 1970 to 28.9/100,000 persons/year in 2014 (5). Twenty percent of women with endometrial cancer are premenopausal, and the standard treatment of endometrial cancer includes bilateral salpingo-oophorectomy. The use of estrogen replacement therapy (ERT) in women with a history of endometrial cancer has been controversial. The fear is that estrogen up-regulates estrogen receptor expression and stimulates growth in endometrial cancer cells (6), but the theory has not been consolidated in clinical studies. A few retrospective studies and case-control studies have been published and have not been able to show increased risk of recurrence after ERT treatment of patients surgically treated for endometrial adenocarcinoma (7–9). The Swedish National Guidelines on endometrial cancer state that the risk of endometrial cancer recurrence after ERT treatment is low or possibly none (10).

Ovarian cancer is the second most common gynecological cancer in Sweden. The incidence has decreased over the last decades from 23.8/100,000 persons/year in 1970 to 14.4/100,000 persons/year in 2014 (5). Even though the incidence increases with age, a significant proportion of cases are diagnosed in premenopausal and perimenopausal women. As for endometrial cancer, physicians have been reluctant to give ovarian cancer survivors ERT because of fear of relapse and decreased survival. Studies have demonstrated a favorable outcome in women treated with HRT following ovarian cancer in comparison to non-users (11–14). The Swedish National Guidelines for ovarian cancer recommend that women with iatrogenic menopause symptoms after primary treatment for epithelial ovarian cancer can be treated with HRT without any known risk of recurrence of disease or decreased survival. Therefore HRT can be offered to women with a history of endometrial or ovarian cancer with moderate to severe menopausal symptoms (10). After hysterectomy, estrogen only should be offered after the assessment of risk factors (15).

Hancke et al. published a survey in 2010 where German gynecologists/gynecologic oncologists were asked about prescribing practice in women with low- or high-risk endometrial cancer and menopausal symptoms (16). In a follow-up study by Yokoyama et al. in 2015, the same survey was used on Japanese gynecologists (17).

The aim with the present study was to illustrate Swedish gynecologists’ and gynecologic oncologists’ attitudes concerning prescription of ERT to women treated for endometrial/ovarian cancer using the same endometrial cancer cases as in the German and Japanese survey studies and to see if prescribing practice is consistent with the available evidence and the Swedish National Guidelines.

## Material and methods

A link to a web-based survey (SurveyMonkey) was distributed to the e-mail membership list of the Swedish Society of Gynecologic Oncology (66 members) in January 2017 and to members of the Swedish Society of Obstetrics and Gynecology (2100 members) in March 2017. A follow-up e-mail was sent 2 weeks later. The survey contained questions regarding gender, age, workplace, and sub-specialization. Respondents were presented with three hypothetical cases (Appendix I) and asked to answer multiple-choice questions regarding ERT. The first case was a 41-year-old woman with FIGO (International Federation of Gynecology and Obstetrics) stage IB grade 2 endometrial cancer and moderate menopausal symptoms. A second case regarded a 38-year-old woman with stage IIIC1 grade 3 endometrial cancer with severe menopausal symptoms. These cases have been featured in previous surveys in Germany and Japan (16,17). In addition, we created one hypothetical case with a stage IIA ovarian cancer patient with moderate menopausal symptoms. The answers were gathered in an anonymous database. The results of the endometrial cancer cases were compared with the results from the German and Japanese studies.

### Statistics

A *p* value was calculated with Fisher’s exact test, and a value of <0.05 was considered to be statistically significant.

### Ethical approval

An application to the ethical review board in Uppsala, Sweden was submitted, but the board found that a permit for this study was not applicable.

## Results

The aim was to distribute the survey to the 2100 Swedish gynecologists registered as members of the Swedish Society of Obstetrics and Gynecology. There was an uncertainty about how many actually received the survey due to doubt regarding the status of the membership e-mail list. Additionally, an e-mail filter in the respondents’ mail system might have sifted out the survey. Therefore, we are unfortunately unable to calculate the actual response rate for the gynecologists. In total, we received answers from 362 gynecologists, out of whom 100 did not complete the survey (reasons being because they never handled these kinds of patients, were retired, etc.). The survey was also distributed to 66 gynecologic oncologists: 28 started the survey, and 4 terminated (residents), yielding a response rate of 38.7% (24/62).

There was no difference regarding distribution of age, gender, and specialization, but a higher proportion of gynecologic oncologists worked at a university hospital (62.5%) compared to gynecologists (33%) (Table 1).

**Table 1.** Characteristics of the respondents.

	Gynecologists		Gynecologic oncologists		*p* value^a^
	*n* = 262	%	*n* = 24	%	
Age <50 years	106	40.1%	12	50%	0.39
Female	191	72.9%	16	66.7%	0.48
Specialization, completed survey (started survey)	262 (362)		24 (28)		
Gynecologic oncologist	30 (30)	11.5%	19 (19)	79.2%	
Tumor surgeon	20 (20)	7.6%	3 (3)	12.5%	
General gynecology	136 (148)	52%	0	0%	
Other (obs/repro)	42 (75)	16%	1 (1)	4.2%	
Resident	20 (74)	7.6%	0 (4)	0%	
No answer of title	14 (15)	5.3%	1 (1)	4.2%	
Work location					0.0062
University hospital	86	32.8%	15	62.5%	
Non-university hospital	130	49.6%	7	29.2%	
Private practice	37	14.1%	1	4.2%	
No answer	9	3.4%	0	0%	

^a^Fisher’s exact test.

### Low-risk endometrial cancer

**Figure 1.**
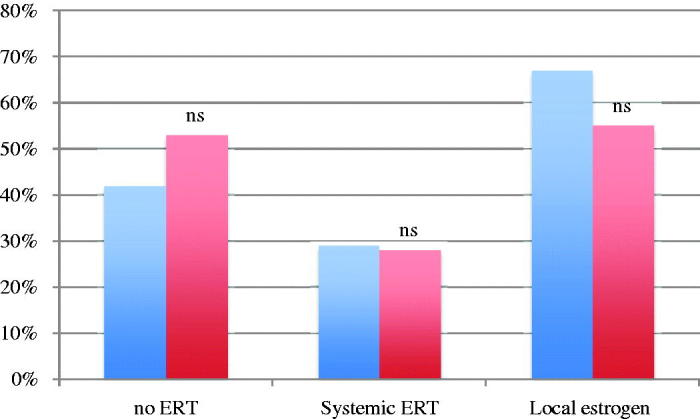
Percentage of gynecologic oncologists (blue) and gynecologists (red) who agree/strongly agree that estrogen replacement therapy (ERT) is contraindicated (no ERT), prescribe systemic ERT or local estrogen therapy. Non-significant differences between gynecologic oncologists and gynecologists (Fisher’s exact test).

In the first case, illustrating a patient with low-risk endometrial cancer and decreased libido, dyspareunia due to vaginal atrophy, and intermittent moderate hot flushes, a total of 53.1% (139/262) of the gynecologists and 41.7% (10/24) of the gynecologic oncologists answered that ERT was contraindicated (Figure 1). A majority of the gynecologists (55%) and gynecologic oncologists (66.7%) would prefer to prescribe local estrogen, whereas 14.9% (39/262) of the gynecologists and 4.2% (1/24) of the gynecologic oncologists would not prescribe estrogen at all (*p* = 0.22). The gynecologic oncologists felt more comfortable treating patients with low-risk endometrial cancer than did the gynecologists (75% versus 42.7%) (*p* = 0.003). Of the gynecologists, 18.7% (49/262) would seek opinion from a more experienced colleague, and 37.0% (97/262) would read the Swedish National Guidelines; the corresponding rates for the gynecologic oncologists were 8.3% (ns) and 16.7% (*p* = 0.047) respectively.

### High-risk endometrial cancer

**Figure 2.**
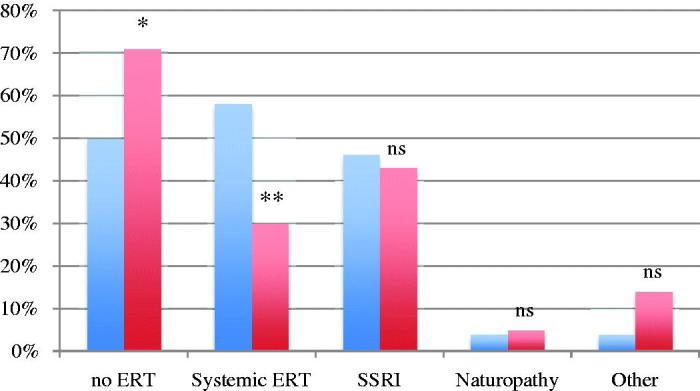
Percentage of gynecologic oncologists (blue) and gynecologists (red) who agree/strongly agree that estrogen replacement therapy (ERT) is contraindicated (no ERT) and drug of choice in the high-risk endometrial case. *p = 0.039; **p = 0.011 (Fisher’s exact test).

The second case was a patient with high-risk endometrial cancer with persistent severe hot flushes and additionally a family history of osteoporosis. A higher proportion thought that ERT was contraindicated in this case, 70.9% (186/262) of the gynecologists and 50% (12/24) of the gynecologic oncologists (*p* = 0.039) (Figure 2). Thirty percent (79/262) of the gynecologists stated that they would prescribe ERT in this case compared to 58.3% (14/24) of the gynecologic oncologists (*p* = 0.011). As non-hormonal alternative 43.1% of the gynecologists would prescribe SSRI as first choice, compared to 45.8% of the gynecologic oncologists. Five percent of the gynecologists would recommend naturopathy instead of ERT, and 14% would choose other drugs. These numbers among the gynecologic oncologists were 4.2% (1/24) for naturopathy and 4.2% for other drugs (ns) (Figure 2).

The gynecologic oncologists felt more comfortable (54.2%) treating patients with high-risk endometrial cancer compared to gynecologists (31.3%) (*p* = 0.039). There was no statistically significant difference between gynecologists and gynecologic oncologists in how many would read the National Guidelines (39% versus 25%) and seek second opinion (26% versus 20.8%).

### Epithelial ovarian cancer

The third case was a patient with stage IIA high-grade serous ovarian cancer suffering from sleeping problems and flushing. She had no known BRCA (BReast CAncer gene) mutation. All of the gynecologic oncologists and 66.4% of the gynecologists stated that ERT was not contraindicated in this case. Sixty-four percent (167/262) of the gynecologists and 91.7% (22/24) of gynecologic oncologists would prescribe ERT (*p* = 0.006). Thirty-nine percent (103/262) of the gynecologists felt comfortable treating the patient compared to 79.2% of the gynecologic oncologists (*p* = 0.0002). In total 22.9% (60/262) of the gynecologists would seek second opinion about treatment compared to 4.2% (1/24) of gynecologic oncologists (*p* = 0.035). Of the gynecologists, 34.7% (91/262) would read the National Guidelines compared to 16.7% (4/24) of the gynecologic oncologists (ns).

In the three hypothetical cases where respondents would not prescribe ERT the most common reason among both gynecologists (62.5%) and gynecologic oncologists (50%) was fear of increased risk of cancer recurrence (ns). Of the gynecologists, 16% said it was because of fear of both cancer recurrence and breast cancer, compared to 8.3% of the gynecologic oncologists.

### Comparison to Japanese and German studies

Hancke et al. published in 2010 a survey study regarding German physicians’ attitude towards prescribing ERT after endometrial cancer. Physicians were asked about their prescribing practice concerning moderate to severe menopausal symptoms for patients having undergone hysterectomy and salpingo-oophorectomy for low-risk and high-risk endometrial cancer. The response rate was 39.8% (165/420) (16). Yokoyama et al. published in 2015 a survey study on Japanese gynecologists’ view on ERT using the same hypothetical cases. In total 880 members of the Japanese Gynecology Oncology group were asked, with a response rate of 40.9% (17). We used the same type of endometrial cancer cases as the two studies, enabling comparisons of results.

The ratio of respondents that would prescribe systemic ERT in the low-risk endometrial cancer case was 43% in Japan, 28.3% in Sweden, and 13% in Germany (Table 2). A majority of respondents in Germany (67%) and Sweden (55.9%) would prefer local estrogen. In Japan, only 15% would prescribe local estrogen, with a higher proportion (43%) giving systemic ERT.

**Table 2.** Comparison of prescription intentions between Swedish, Japanese, and German doctors.

	Sweden		Japan		Germany	
	Low-risk	High-risk	Low-risk	High-risk	Low-risk	High-risk
Number (*n*)	286	286	363	363	165	165
ERT not contraindicated	47%	28%	65%	49%	46%	25%
Systemic ERT	28%^a^^,b^	33%^c^	43%^a^	37%	13%^b^	18%^c^
Local ERT	56%		15%		67%	
SSRI		43%^d^		0%		29%^d^
Phytoestrogen/naturopathy		5%	2%	3%		45%
Second opinion	18%	26%	2%	3%	36%	50%

Comparison of prescription intention between Swedish, Japanese, and German respondents for ERT in low-risk versus high-risk endometrial cancer cases.

^a^*p* < 0.00001; ^b^*p* = 0.00016; ^c^*p* = 0.0007; ^d^*p* = 0.004 (Fisher’s exact test).

In the high-risk endometrial cancer case 33% in the Swedish study would prescribe ERT in comparison to 37% in Japan (ns) and 18% in Germany. In Japan 50% of respondents would choose traditional Japanese drugs, and none would prescribe SSRI. Responders in Sweden and Germany seek second opinion more often than their colleagues in Japan.

## Discussion

Prescribing ERT to women with a history of ovarian/endometrial cancer is a difficult decision. Based on this survey many of the respondents had a favorable attitude towards ERT after treatment of epithelial ovarian or endometrial cancer. The gynecologic oncologists had a more favorable attitude than the gynecologists in the high-risk endometrial cancer case and the ovarian cancer case.

The gynecologic oncologists were members of the Swedish Society of Gynecologic Oncology, and according to The National Board of Health and Welfare a total of 62 gynecologic oncologists were professionally active in 2015 (18). Consequently, the questionnaire reached almost all gynecologic oncologists in Sweden. The response rate was fairly low (38.7%) but similar to those in the Japanese (40.9%) and German (39.8%) survey studies. A problem appeared when sending the survey to the gynecologists because of a possible mass e-mail filter in the responders’ mail system. In fact, it might have sifted out the survey, leading to an uncertainty in the number of members who actually received the mail, and this made it impossible to calculate a true response rate (262/2100). A possible bias may appear if those who choose to respond to the survey have more knowledge or experience of the subject, resulting in a false high frequency of adherence to National Guidelines. Another potential problem might be that respondents may not give accurate answers or feel comfortable providing answers that present themselves in an unfavorable manner even though the study is anonymous.

In the low-risk endometrial cancer case, the Japanese respondents were the most likely to prescribe ERT more often than the Swedish and German participants. In the high-risk case Swedish and Japanese respondents were more likely to prescribe HRT than Germans. The Japanese study was published in 2013 whereas the German one was published in 2006, which might influence the respondents’ attitude towards HRT.

Concerns regarding HRT in postmenopausal women with no history of gynecologic cancer are that it may increase the risk of venous thromboembolic events (19), cerebrovascular accidents, breast cancer, and coronary heart disease (CHD). The prescription of HRT increased from the beginning of the 1980s until the late 1990s but decreased dramatically after publication of e.g. the Women’s Health Initiative study (WHI) in 2002, where results showed that HRT increased the risk of CHD and breast cancer in postmenopausal women (20). Subsequent re-analyses of data from the WHI study now consistently show reductions in CHD and mortality when HRT is initiated close to menopause (2). HRT with estrogen and progesterone can be associated with an increase in the risk of breast cancer, with an additional 8 cases diagnosed per 10,000 women over 5 years. The risk is related to treatment duration and reduced after stopping HRT (21). However, data from the WHI study showed that treatment with estrogen alone among women with prior hysterectomy did not increase the risk of breast cancer, CHD, or all-cause mortality (22).

HRT use in Sweden in the age group 50–59 years decreased from a peak of 36% in 1999 to 9% in 2007 (information from national pharmacy data) (23), and according to The Swedish National Board of Health and Welfare it was almost unchanged in 2015 (8.8%) (24). Retrospective studies of postoperative HRT for endometrial cancer (mostly stage I–II) show no significant differences in cancer recurrence or survival of women who take HRT (7–9). Barakat et al. published in 2006 the only randomized study on ERT after early-stage endometrial cancer treatment. The study included 1236 patients randomized to receive either ERT or placebo after cancer treatment during 1997–2003. The study was stopped early because of difficulties recruiting patients after the results of the WHI study 2002. Because of lack of study power they could not conclusively support or disprove the safety of ERT with regard to risk of endometrial cancer recurrence, but the absolute recurrence rate (2.1%) was low when 55% of the patients had been taking estrogen for more than 2 years (8).

Guidozzi and Daponte published in 1999 a randomized controlled study about ERT in epithelial ovarian carcinoma survivors: 130 patients <59 years old treated for invasive epithelial ovarian cancer were randomized for ERT versus placebo and followed for 48 months. The study could not show any difference in disease-free and overall survival between groups (12). A Swedish prospective cohort study of 649 patients with ovarian cancer where 150 patients received HRT after primary treatment showed a better survival among women who used HRT after diagnosis (11). In 2015 Eeles et al. published an article on HRT effect on survival and disease outcome in women with epithelial ovarian cancer. In total 150 ovarian cancer patients were randomized to HRT versus placebo in 1990–1995. Overall survival was improved in the HRT group after follow-up for 19 years (14). A meta-analysis published in 2015 suggests that HRT use by women with a history of epithelial ovarian cancer did not lead to a significantly increased risk of death or recurrence of disease (25).

Non-hormonal management of postmenopausal symptoms includes lifestyle modification, diet, and application of behavioral and alternative medicine therapies, though the results of the impact of these treatments are conflicting.

The Swedish National Guidelines on epithelial ovarian and endometrial cancer state that ERT can be used in survivors in selected cases. As mentioned, HRT is the most effective treatment for vasomotor symptoms associated with menopause. Benefits such as positive effects on menopausal symptoms as well as the protective effect on the cardiovascular disease and osteoporosis have to be considered and are likely to outweigh risks for symptomatic women before the age of 60 years or within 10 years after menopause. The risk and benefit of HRT should thus be individualized for every woman in premature menopause.

The results from the present study demonstrate a sometimes unjustified fear of using ERT in endometrial/ovarian cancer survivors and that the Swedish National Guidelines are not properly followed. Those more experienced with gynecologic cancer patients (gynecologic oncologists) have a more favorable attitude towards ERT for endometrial/ovarian cancer patients than do general gynecologists, and they feel more comfortable treating their patients. This study illustrates a need for more education on these matters. Quality of life as well as other benefits with HRT should continuously be addressed in the follow-up visits. More studies regarding the benefits and risks of HRT in gynecologic cancer survivors are needed and might be possible with improved pharmaceutical registers.
